# Arctic Mosquitoes

**Published:** 1882-11

**Authors:** 


### ARCTIC MOSQUITOES.

In a work recently published in London, the author, Edward Roe, gives the following account of the Arctic mosquitoes, which almost makes us content with our own :

"The one bitter drop in our cup of joy was the monstrous but inseparable curse of Arctic summer life—the mosquito. He abounded, flourished, luxuriated, surpassed himself, out-mosquitoed himself, on the Kuloi River. We were at his mercy ; our veils, gauntlets, handkerchiefs, flapper, all were a vanity and vexation. To kill was wanton, for to destroy sufficient was impossible. We had foreseen all this, and had even thought of taking, among other things, a woodpecker from home with us to protect our faces while we slept ; but one woodpecker would have been a solemn mockery ; we should have wanted a fresh woodpecker every five minutes. I suppos these were the historical flies sent to punish the disobedient, obstinate Egyptians; they came forth in order, and after three grievous plagues—the corruption of the waters, the multitude of frogs, and the swarms of lice— had entirely failed.

"We are becoming connoisseurs in mosquitoes; we watch them traverse our veils like figures on slides in a magic lantern. There is the yellow-striped vampire mosquito, with a triple fang to his proboscis; there is the brown, hump-backed or camel mosquito, with legs of gossamer, who appears to our vindictive eyes to be from two to three inches in length; finally, there is the scorpion-mosquito, very searching and business-like. We dislike him greatly, for he wastes no time. We know now that leather is a hollow delusion, and armor-plated gauntlets are alone of avail.

"Sometimes a mosquito comes and kills himself by squeezing between our finger and thumb, sometimes by flying against my flapper. There are moments, but so rare and delicious that I almost tremble to describe them, when we find a mosquito who has anchored himself by the proboscis in our gloves—and we watch the expression of baffled hatred in his countenance with which he watches the approach of the avenging finger. O, the peaceful, blissful enjoyment of that moment! Sometimes we watch him in his anxious, hurried efforts to pierce the glove—he knows that time is all he needs—standing upon his fore legs, with his hind legs flourishing in the air, while he bores away diligently through the thick leather in his wicked thirst for blood. Sometimes in our frenzy we ensnare a mosquito and get up and trample on his head. We ask ourselves in hours past endurance why the law of nature should be reversed, and Man, the lord of creation, become the prey of savage creatures. We have formed a grave if impious resolution; we will take a mosquito by stratagem, pinion him, and, with the help of a burning glass, offer him in sacrifice to the Midnight Sun."

---

NEVER forsake a friend. When enemies gather around—when sickness falls on the heart—when the world is dark and cheerless—is the time to try true friendship. They who turn from the scene of distress, betray their hypocrisy, and prove that interest only moves them. If you have a friend who loves you, who has studied your interest and happiness—be sure to sustain him in adversity. Let him feel that his former kindness is appreciated, and that his love was not thrown away. Real fidelity may be rare, but it exists—in the heart. They only deny its worth and power who have never loved a friend, or labored to make one happy.

